# Hybridization selects for prime‐numbered life cycles in *Magicicada*: An individual‐based simulation model of a structured periodical cicada population

**DOI:** 10.1002/ece3.6270

**Published:** 2020-04-29

**Authors:** Jaakko Toivonen, Lutz Fromhage

**Affiliations:** ^1^ Department of Computer Science University of Helsinki Helsinki Finland; ^2^ Department of Biological and Environmental Science University of Jyväskylä Jyväskylä Finland

**Keywords:** individual‐based model, *Magicicada*, prime numbers, structured population model

## Abstract

We investigate competition between separate periodical cicada populations each possessing different life‐cycle lengths. We build an individual‐based model to simulate the cicada life cycle and allow random migrations to occur between patches inhabited by the different populations. We show that if hybridization between different cycle lengths produces offspring that have an intermediate life‐cycle length, then predation acts disproportionately to select against the hybrid offspring. This happens because they emerge in low densities without the safety‐in‐numbers provided by either parent population. Thus, prime‐numbered life cycles that can better avoid hybridization are favored. However, we find that this advantage of prime‐numbered cycles occurs only if there is some mechanism that can occasionally synchronize emergence between local populations in sufficiently many patches.

## Graphical abstract

We investigate competition between separate periodical cicada populations each possessing different life‐cycle lengths. We show that prime‐numbered life cycles are selected for when hybridization produces offspring with an intermediate life‐cycle length compared to their parents.

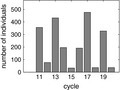

## 1 INTRODUCTION

Periodical cicadas (*Magicicada* spp.) are famous for their long, synchronous, and prime‐numbered life cycles. The ecology and evolution of the periodical cicadas have been studied extensively (Alexander & Moore, 1962; Dybas & Davis, 1962; Dybas & Lloyd, 1962, 1974; Lloyd & Dybas, 1966a, 1966b; Marshall & Cooley, 2000; White & Lloyd, 1975, 1979) with a general review given by Williams and Simon (1995). Previously, it has been suggested that the periodical cicadas evolved from nonperiodical cicadas by switching from a size‐based to an age‐based emergence strategy (Yoshimura, 1997). Here, size‐based emergence refers to an emergence strategy, where an individual emerges from the ground to mate immediately upon reaching some threshold body size. Age‐based emergence refers to an emergence strategy where an individual waits for a fixed number of years before emergence. In a nonperiodical cicada population, individuals have variable life‐cycle lengths and typically some individuals emerge each year from the ground to mate. Periodical cicada populations on the other hand display a life cycle where all individuals stay in the ground for a fixed number of years and then emerge synchronously at the end of their life cycle. The large numbers of individuals emerging simultaneously have been thought of as a mechanism to reduce per capita mortality due to predation, as in a large crowd the probability of any given individual to be eaten is small (Karban, 1982).

The switch from size‐ to aged‐based emergence has been shown to be plausible by using individual‐based models (IBM) to simulate the cicada life cycle and population dynamics (Ito et al., 2015). Further, it has been shown that this switch can in principle result in any cycle length evolving, and that the particular cycle length that does evolve depends on the abiotic environmental conditions and on the relative strength of predation affecting cicada emergence (Toivonen & Fromhage, 2018). However, recently it was shown that evolutionary hysteresis and ratchet effects can result in progressively longer cycle lengths evolving if the abiotic environment undergoes periods of progressively harsher conditions similar to those experienced during the Pleistocene ice age (Toivonen & Fromhage, 2019). Phylogenetic studies of *Magicicada* suggest that the origin of the *Magicicada* genus was before this time, but the split to current 13‐ and 17‐year cycles occurred during the Pleistocene ice age circa 0.5 Mya (Sota et al., 2013). Thus, it has been suggested that *Magicicada* developed proto‐periodicity (where emergence is not fully synchronous but shows a repeating pattern of high and low emergence density years) or possibly even full periodicity before the Pleistocene ice age, but their life cycles later became longer due to the harsh environmental conditions of glacial periods (Toivonen & Fromhage, 2019).

This previous work has not, however, addressed the question of why *Magicicada* life cycles are prime‐numbered. One of the mechanisms suggested to have favored prime‐numbered cycle lengths in the evolution of *Magicicada* is hybridization (Cox & Carlton, 1988; Yoshimura, 1997). This theory assumes that mating between individuals of different life‐cycle lengths produces offspring that have a life‐cycle length different from the parent cycles. Then, the offspring have low viability as they emerge without the safety‐in‐numbers provided by emerging with either parent population and are thus heavily predated on. At the same time, both parent populations suffer from decreased population density as a significant proportion of their offspring are lost. As a result of the fitness costs of hybridization, it has been suggested that prime‐numbered cycles would be advantageous because they coemerge with other cycles less frequently than nonprime‐numbered cycles do (Cox & Carlton, 1988, 1998; Yoshimura, 1997). However, this is not necessarily true, for instance as any two even‐numbered life cycles never coemerge when one life cycle emerges on odd years, and the other on even years (Lehmann‐Ziebarth et al., 2005).

It is likely that during glacial periods many different cicada communities existed in isolation (Sota et al., 2013) and previous theoretical work suggests that each of these communities could have evolved a distinct life‐cycle length (Toivonen & Fromhage, 2018, 2019). During interglacials, these populations would likely have come in contact with each other again. In this paper, our goal is to investigate whether hybridization between populations of periodical cicadas with differing cycle lengths can favor prime‐numbered cycle lengths. We use an individual‐based model to simulate cicada life cycles in a structured population that consists of patches inhabited by periodical cicada populations with differing cycle lengths. We show that prime‐numbered cycles are favored, if every local population at the start of a simulation consists of newborns only. In this scenario, prime‐numbered cycles coemerge with other cycles less often than nonprime‐numbered cycles do, due to the properties of prime numbers. Tanaka, Yoshimura, Simon, Cooley, and Tainaka (2009) found similar results under certain conditions in a linear, deterministic population model for a single patch. However, the initial population age structure in this scenario is a strictly special case (and it appears to be the only case Tanaka et al. considered). We show that generally prime‐numbered cycles do not coemerge less often than nonprime‐numbered cycles and thus they do not appear to have any discernible advantage. However, we further show that prime‐numbered cycles are indeed favored, if there exists some environmental mechanism that can occasionally cause a sufficient proportion of the whole population to become temporarily synchronized. In particular, it has been observed that some insect species may enter into prolonged dormancy during times of harsh environmental conditions (Danks, 1987), and this dormant period may last for several years followed by a synchronized emergence of the population (e.g., see Powell, 1989). Population‐wide delays in emergence can then effectively set the population emergence schedule sufficiently close to the special case in which prime‐numbered cycles are favored.

## 2 MODEL

Periodical cicadas live most of their life underground as nymphs. Each individual lives in a feeding cell that has access to nutrition from root xylem fluids (Williams & Simon, 1995). The availability of feeding cells is potentially a limiting factor on the size of the cicada population and nymphs compete for occupancy of the feeding cells (Lloyd & Dybas, 1966a; White, Lloyd, & Zar, 1979). In the early summer of the last year of their life cycle, all individuals in a periodical cicada population emerge above ground to mate. During emergence, the cicadas are heavily predated on (Williams & Simon, 1995). After reproduction, the adult cicadas die and the offspring burrow underground, thus beginning a new life cycle.

We use an individual‐based model (IBM) to simulate the yearly cicada life cycle. Each individual has five attributes that are kept track of: two alleles, a phenotype, body size, and age. An individual's phenotype determines whether it follows the size‐ or age‐based emergence strategy. In the former case, an individual waits until it has reached some threshold body size and then emerges the following summer. In the latter case, the phenotype determines how many years an individual will wait before emergence. However, in order to emerge, an individual with the age‐based emergence trait needs to have reached both the threshold body size and the phenotypic minimum age for emergence, that is, the individual waits until it has fulfilled both emergence criteria and then emerges at the first opportunity (Toivonen & Fromhage, 2019). The two different emergence traits are part of the general model previously developed for the evolution of periodic life cycles, wherein age‐based emergence originally evolved from size‐based emergence (Ito et al., 2015; Toivonen & Fromhage, 2018, 2019). However, size‐based emergence will turn out not to be particularly relevant in the context of this paper, in which the starting point is that age‐based emergence has already evolved in multiple isolated habitats. Nevertheless, to be consistent with previous work, we include size‐based emergence as a possible emergence trait.

We assume diploid genetics with one locus and multiple allele variants. One allele variant codes for size‐based emergence and the others for age‐based emergence with a given fixed‐cycle length. We assume that size‐based emergence is dominant, that is, if an individual possesses a size‐based emergence allele, then its phenotype is to emerge according to the size‐based trait. If an individual has two alleles coding for age‐based emergence, then the phenotype corresponds to age‐based emergence with a cycle length that is the average of the allele cycle lengths. If the average is not an integer, then it is rounded randomly up or down to the nearest integer. We note that the choice of dominance is not expected to be significant, since we already begin with all subpopulations having the age‐based emergence trait: when the majority of a given local population is periodic, size‐based emergence is selected against as it may lead an individual to emerge before the main cohort and thus experience an elevated risk of predation (see Toivonen & Fromhage, 2018, 2019 for further discussion).

Each simulation begins with creating the initial cicada nymph population. The population consists of 11 distinct patches each inhabited by a local population possessing the age‐based emergence trait with a distinct cycle length. The existing cycle lengths go from 10 to 20 years so that each of the cycle lengths exists in precisely one patch. This setting allows us to conduct an in silico experiment of fair competition between different cicadas life cycles. The cycle lengths were chosen such that they consist of a group of relatively long cycles that encompass the 13‐ and 17‐year cycles exhibited by *Magicicada*. This is in accordance with the hypothesis that cicada life cycles had become relatively long before the current prime‐numbered cycles were selected for. The population in each patch consists of identical homozygote individuals with the same age and a body size corresponding to the expected mean body size at that age. While all the individuals in a given patch have the same age at the beginning of a simulation, individuals in different patches may or may not have different ages (see Section 3).

**FIGURE 1:**
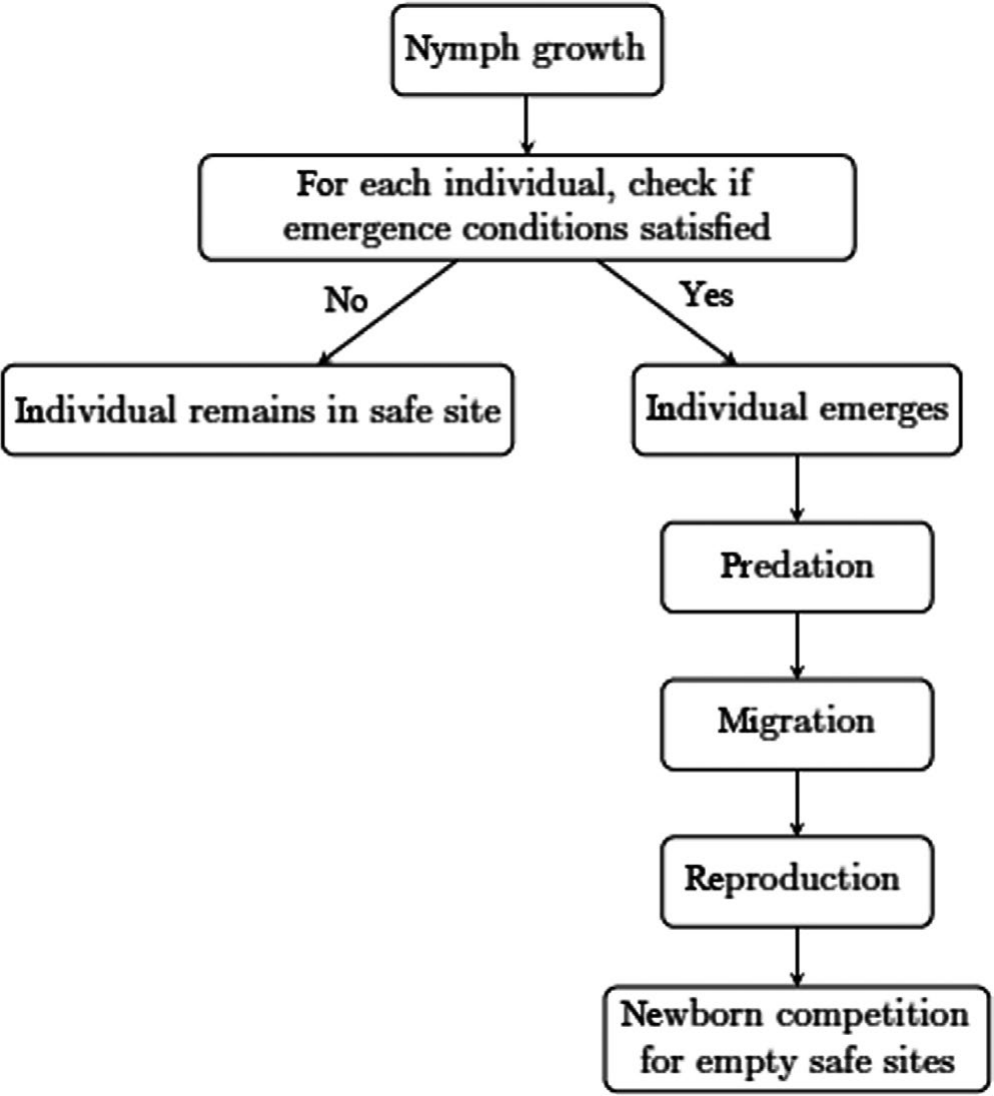
Simulation flowchart modeling *Magicicada* yearly life cycle

A schematic of the model for the yearly cicada life cycle is shown in Figure 1. The first step in the main simulation loop is nymph growth. For each nymph, we increase age by one and body size by a random positive increment drawn from a log‐normal distribution $X=\exp(N\left(\mu ,\sigma^{2}\right))$ , where *µ* is the mean and σ the standard deviation of the normal distribution. This models exponential growth during an annual growth season (typically spring and summer in seasonal climates) with normally distributed individual growth rates. We set the mean growth rate high enough so that age‐based emergence traits with a cycle length greater or equal to 10 will have virtually all individuals reaching the target body size in time before emergence, which is necessary for the traits to remain periodic with synchronous emergence. This also reflects the assumption that the environmental conditions are more favorable now than in the past, when the periodic emergence traits originally evolved (Toivonen & Fromhage, 2019). Once individuals have reached the target body size they do not grow further (or at least further growth is assumed to be irrelevant). We note that it is possible to also think of growth in more abstract terms than merely body size, but rather as measuring development toward reproductive maturity.

Individuals who have fulfilled their emergence conditions are moved from their local nymph population to a local adult population specific to their patch. Then, the adult population emerges from underground and is subjected to predation. Predator induced mortality is assumed to be dependent on the density of the emerging cicada population, but predator density is the same for each patch. We assume a predator‐prey model, where predation follows a Holling type II functional response, which models predator satiation (Holling, 1959; Table A1). More specifically, we numerically solve the equation $\dot{n}=-\left(apn\right)/\left(1+ahn\right)$ for one (arbitrary) time unit, where *n* is the density of adult cicadas emerging in a given patch, *p* is the density of predators, *a* is the predator attack rate and *h* is the handling time for a predator to process a caught prey before it resumes predation. We estimate the probability of a given cicada to survive predation as n(1)/n(0) , where n(0) is the total density of emerging adult cicadas and n(1) is the density remaining after one (arbitrary) time unit. We perform a biased coin toss (i.e., a Bernoulli trial) for each emerging adult based on the patch‐specific survival probability and remove each individual determined to not having survived. In general, the bigger the local adult cicada population, the smaller the probability of a given individual being eaten. If the locally emerging adult population is too small, it may perish entirely.

The surviving adults begin to look for mating opportunities. We assume that during this time there is a fixed probability that any given individual may stray away or otherwise migrate from their own patch to another one. If an individual ends up migrating, we randomly select a new patch for that individual (the original patch is included). Then, every adult individual reproduces. We keep track of female individuals only and assume that sufficiently many males exist in the local population with genotype ratios identical to the female population. Each female mates with a randomly chosen male, the number of offspring is fixed and each newborn receives one randomly chosen allele from both parents. We assume a fixed probability that a given allele may mutate. If a mutation occurs to an age‐based emergence allele, we assume that there is an even chance that the new allele either codes for size‐based emergence or for age‐based emergence with a different cycle length (equal probability to be either one year longer or shorter). If a size‐based allele mutates, we assume that the new allele codes for age‐based emergence with a random cycle length between one and 30.

After reproduction, all the adults die and the newborns compete to find empty feeding cells underground. We assume that newborns cannot establish themselves in feeding cells already occupied by older nymphs. Further, we assume that each newborn finds a feeding cell randomly so that the number of newborns competing for a given feeding cell follows the Poisson distribution. For reasons of computational efficiency, we do not keep track of feeding cells explicitly, but rather use a mechanistically derived formula to approximate the establishment probability of newborns in a given patch. More precisely, we assume that all newborns in a given patch have an equal probability of establishment and this is given by

(1) $$P_{\text{est}}=\left\{\begin{array}{cc} \left(1-\frac{N_{1}}{N}\right)\sum_{k=0}^{N_{0}-1}e^{-N_{0}/N}\frac{{\left(N_{0}/N\right)}^{k}}{\left(k\right)!}\frac{1}{k+1} & ,\;\text{if}\;N_{1}\leq N\\ 0 & ,\;\text{if}\;N_{1}>N. \end{array}\right.$$

where $N_{1}$ is the number of older nymphs already established in the patch, *N* is the total number of feeding cells in the patch (we assume *N* is the same for each patch), and *N*_0_ is the total number of newborns arriving to the patch. Then, $1-N_{1}/N$ is the fraction of empty feeding cells and $N_{0}/N$ is the average number of newborns that compete for a given feeding cell. The term $\exp\left(-N_{0}/N\right){\left(N_{0}/N\right)}^{k}/k!$ gives the probability that exactly *k* competitors end up in the same feeding cell as the focal individual and the term 1/(k+1) gives the probability that the focal individual wins the competition for the feeding cell against *k* competitors. When determining newborn success in establishing themselves in the patch, we perform a biased coin toss with probability of success*P*_est_ for each individual. We emphasize that Equation (1) is only used to calculate an approximate density‐dependent establishment probability for the newborns. In the actual simulation, because we determine success or failure independently for each individual, it is possible for more than *N* nymphs to become established underground. This may be interpreted such that nymphs can temporarily fit more than *N* individuals underground and the number of feeding cells is not a hard cap on population size. However, if there are more than *N* nymphs already established underground before newborns enter, we then assume that the establishment probability of newborns is zero until the nymph population decreases below *N*. Then, typically nymph population density in a given patch varies stochastically around the carrying capacity *N*.

We begin each simulation with a 2000 round (one round corresponds to one year in the cicada life cycle) interval during which migration between patches is not allowed and no mutations occur. Also, during the first 1,000 years predator density is incrementally increased from zero to some positive predefined value. This initial phase allows some time for the resident population in each patch to smoothly settle into a population dynamical equilibrium, which then serves as a natural starting point for the full simulation.

Table A1 shows model parameters and their typical values. Codes and simulation data for the IBM are available at a Dryad repository (Toivonen & Fromhage, 2020).

## 3 RESULTS

### 3.1 Coemergence probabilities

**FIGURE 2:**
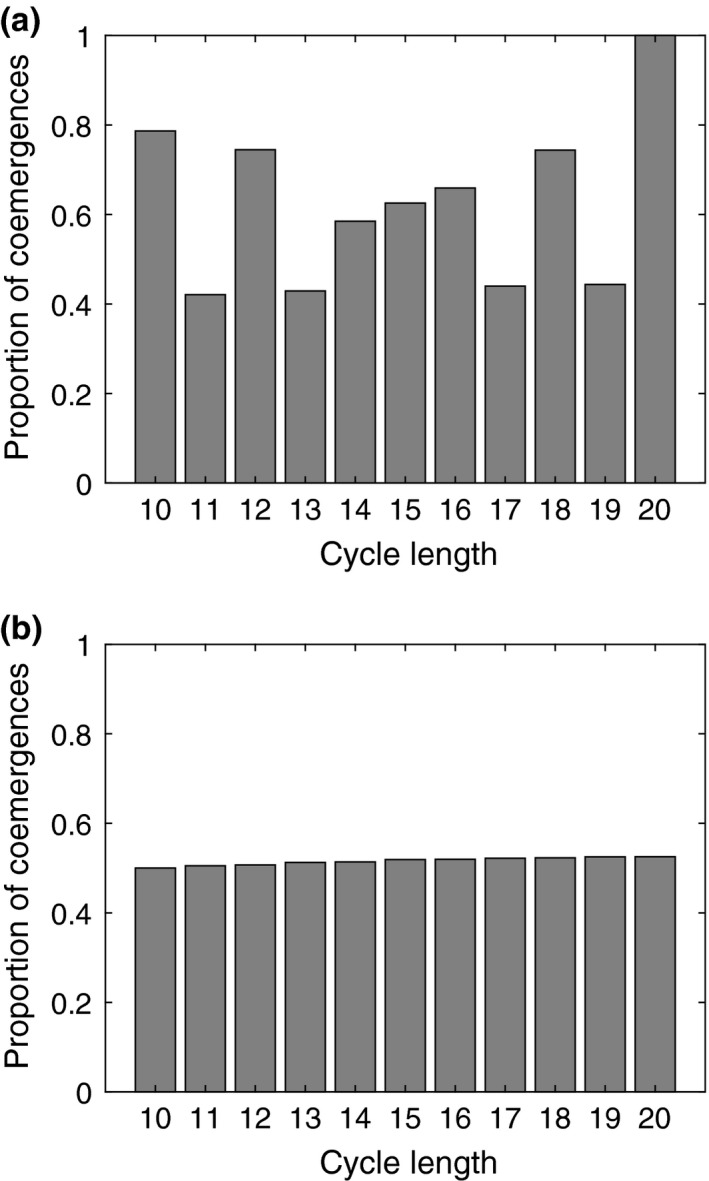
Results for coemergence trials without delays in emergence. (a) Proportion of coemergences in the first 50,000 years, when the starting age of each cycle is zero. (b) Proportion of coemergences, when the starting age of each cycle is random. Here, the proportions are calculated from 10,000 independent trials with a 50,000 year time interval in each trial

We do not need the full IBM simulations to already gain certain insights about coemergence probabilities of different cycle lengths. We take all cycle lengths between 10 and 20, assign a starting age for each cycle and then map out precisely when each cycle emerges in the future (assuming each cycle always emerges perfectly on time). If each cycle has age zero at the start of our observation interval, then we see that prime‐numbered cycle lengths coemerge less frequently than nonprime‐numbered cycles (Figure 2a). Next, we perform multiple trials with randomly chosen initial ages for each cycle and observe that the average proportion of coemergences is now practically the same for each cycle (Figure 2b). In particular, whether a cycle length is prime or not, appears to have no discernible effect on the proportion of coemergences. As we perform multiple trials where we map out emergence over long time intervals, the average proportion of coemergences (calculated over all the trials) serves as a general measure for the probability of coemergence for each cycle.

**FIGURE 3:**
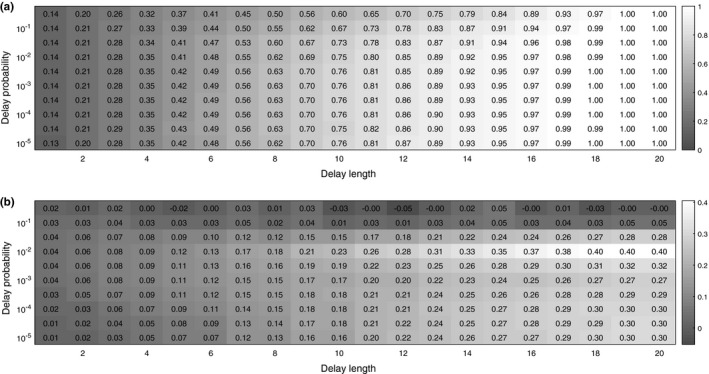
Results for coemergence trials with random delays in emergence. Note the logarithmic scale on the vertical axis. (a) Average proportion of cycles that coemerge immediately after a delay. (b) Difference in mean probability of coemergence between nonprime and prime‐numbered cycles, recorded from the first delay onwards

Next, we allow a constant yearly probability $p_{\text{delay}}$ such that no emergence can occur for the next *d* years (and we assume new delays may not occur during a delay). After *d* years have passed from the start of the delay, all cycles that have reached their emergence age do emerge. We again choose random starting ages for each cycle, map out their future emergence years, randomly distribute delays across the observation time interval and move emergence years accordingly for each cycle that would have otherwise emerged during a delay interval. Then, we collect statistics on coemerge probabilities for different combinations of*p*_delay_ and *d* (Figure 3).

First, we measure how much delays synchronize the entire population as a whole, that is, we look at the average proportion of cycles that emerge right after a delay interval has ended (Figure 3a). As might be expected, long delays (high *d*) lead to full synchronization of the whole population, that is, once a delay ends all cycles emerge on the same year. Conversely, small delays (low *d*) result in a small proportion of the cycles to coemerge. In general, as delay length increases, so does the level of synchronization. However, it is important to note that even fairly large delays (e.g., *d *= 10) do not lead to full synchrony of the entire population.

Next, we measure how different values of*p*_delay_ and *d* affect the difference between mean coemergence probabilities for nonprime and prime‐numbered cycles. We see that if the delay length *d* is very small, then this difference is small. However, if*p*_delay_ is not very low or very high (i.e., $10^{-5}<p_{\text{delay}}<10^{-1}$ ), then the difference in mean coemergence probabilites between nonprime and prime‐numbered cycles increases as *p*_delay_ increases. When the delay probability *p*_delay_ becomes very small, it can happen that no delay occurs during a finite observation time interval. If we only count mean proportion of coemergences from trials where at least one delay occurs and only look at emergences after the first delay, then difference in coemergence probabilities remains high even when the delay probability becomes very small (Figure 3b).

Finally, we note that even though very frequent delays ( $p_{\text{delay}}\geq 10^{-1}$ ) result in high synchronization (Figure 3a), this does not lead to a large difference between coemergence probabilities of nonprime and prime‐numbered cycles (Figure 3b). This is because very frequently occurring delays mean small time windows for emergence, and often the timing of emergence is determined by the end of a delay rather than the intrinsic emergence schedule of a given cycle. In contrast, when delays do not occur very frequently ( $p_{\text{delay}}<10^{-1}$ ), then synchronization leads to an increased difference in coemergence probabilities of nonprime and prime‐numbered cycles.

Codes pertaining to the calculations in this section are available at a Dryad repository (Toivonen & Fromhage, 2020).

### 3.2 Full IBM simulations

**FIGURE 4:**
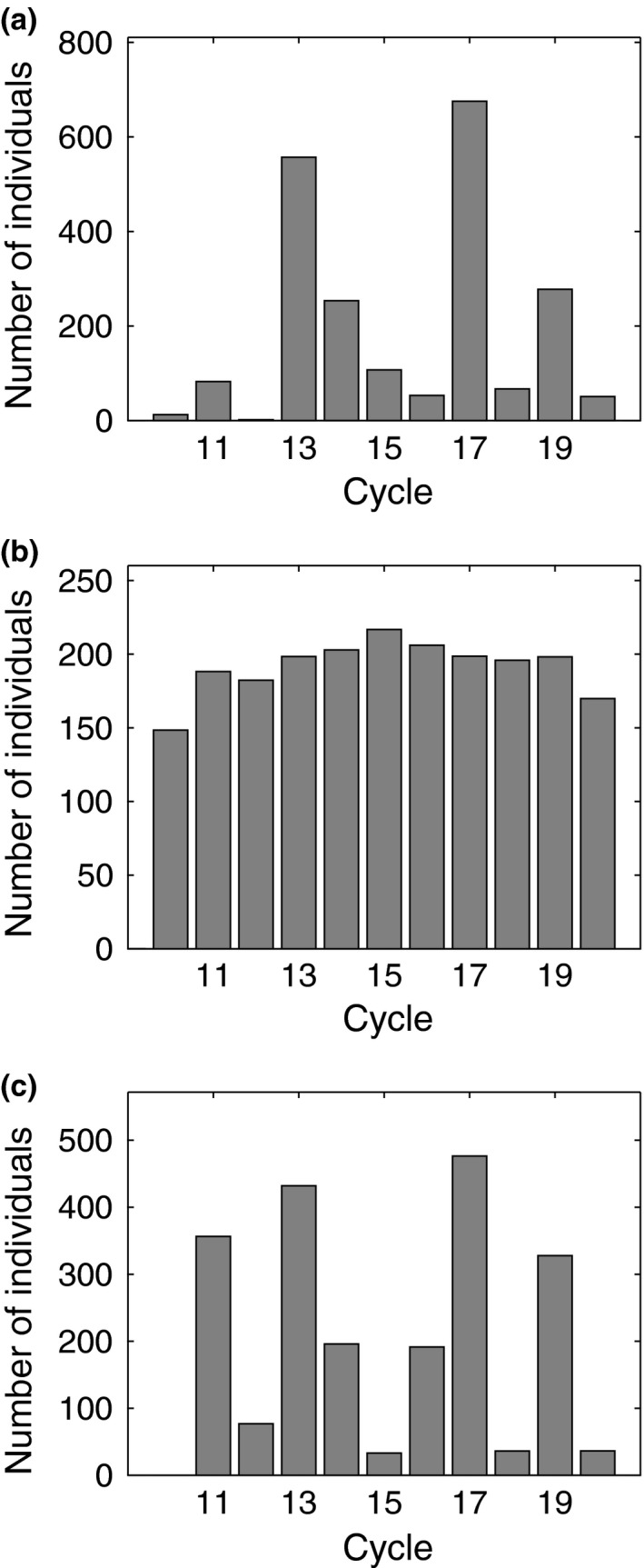
Average number of individuals for each cycle length at the end of the IBM simulations. We run 30 independent simulations for each scenario and each simulation was run for 50,000 rounds (years) after the 2,000 round initialization phase. (a) Each subpopulation consists of newborns only at the start of the simulations. At the end of the simulations, the proportion of prime‐numbered cycle individuals is 74%. Twenty simulations ended with one cycle length having invaded the whole population with all other cycles extinct. (b) Each subpopulation has a randomly drawn age at the start of the simulations. At the end of the simulations, the proportion of prime‐numbered cycle individuals is 37%. Almost all cycles remain dominant in their respective original patches. (c) Each subpopulation has a randomly drawn age at the start of the simulations. In this scenario, we include an annual probability of 0.0001 that a 20‐year delay in emergence occurs. At the end of the simulations, proportion of prime‐numbered cycle individuals is 74%. Twenty‐four simulations ended with one cycle length having invaded the whole population with all other cycles extinct

First, we perform 30 independent simulations of the model while assuming that the local populations in each patch consist of only newborns. As expected based on the results on coemergence probabilities, we find that prime‐numbered cycles are heavily favored in this scenario (Figure 4a). More specifically, we find that the average density of prime‐numbered cycles at the end of the simulations is about 74% of the entire population while at the beginning of the simulations their proportion was only 4/11 ≈ 36%. The majority of the simulations end with virtually all individuals across all patches synchronous and having the same fixed life‐cycle length.

Next, we run 30 independent simulations of the model while assuming that each subpopulation has a randomly chosen starting age between 0 and the threshold emergence age of that particular subpopulation. Again, as expected based on the results on coemergence probabilities, we do not find any particular advantage for prime‐numbered cycles (Figure 4b). More specifically, the average density of prime‐numbered cycles at the end of the simulations is about 37% of the whole population, while their proportion of the initial population is 4/11 ≈ 36%. Contrary to the case when the entire initial population consisted of newborns only, here we notice that local populations tend to be quite stable against invasion from other cycles. More specifically, all simulations end with at least nine of the original cycles holding their initial patch.

Next, we conduct simulations where we assume again that the initial age of each subpopulation is random, but we allow a small probability each year that such adverse weather conditions occur so as to entirely prohibit the emergence of cicadas across all the patches. Just as before, we again assume a constant probability *p*_delay_ such that no emergence can occur for the next *d* years. Otherwise, the model is as before so that nymphs keep growing during this interval, but emergence is not possible (or development to maturity is suppressed). After *d *years have passed from the start of the delay, all individuals who have reached their emergence condition do emerge.

**FIGURE 5:**
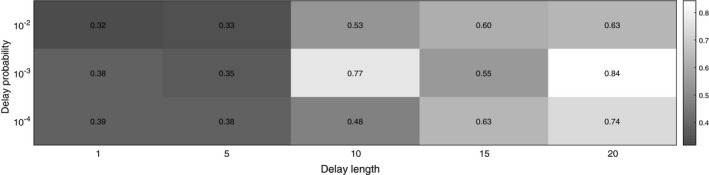
Average proportion of individuals with a prime‐numbered cycle at the end of simulations of the full IBM for different combinations of delay length and probability. Thirty independent simulations were performed for each combination of delay length and probability. The initial age for each subpopulation was randomly chosen. Note the logarithmic scale on the vertical axis

In line with the results on coemergence probabilities, we do not find a significant change in the results, if the delay *d* is small. However, prime‐numbered cycles become more favored with sufficiently long delays, when $p_{\text{delay}}\leq 10^{-2}$ (Figure 4c): for example, the average proportion of prime‐numbered cycles (across $p_{\text{delay}}=\left\{10^{-2},10^{-3},10^{-4}\right\}$ ) at the end of the simulations was 59% of the whole population with d=10 and 74% with d=20 (Figure 5). With $p_{\text{delay}}=10^{-1}$ we find that prime numbers are not favored per se, but when the delay length d>10 , there appears to be an advantage for cycle lengths close to *d* (see Appendix 1 for further discussion).

**FIGURE 6:**
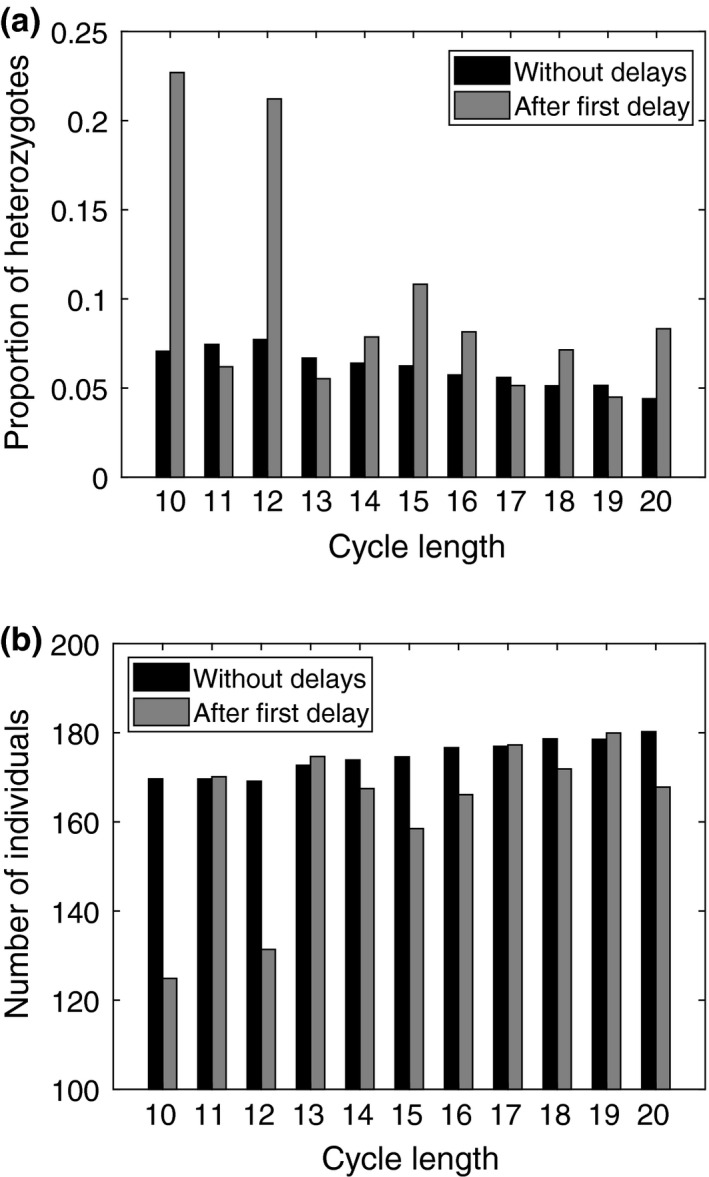
Results from 30 independent simulations either with or without delays. Each simulation begins with randomly chosen initial subpopulation ages. Horizontal axis indicates the original cycle length in each patch. Black bars indicate averages for the case without delays and gray bars for averages after the first delay (in the case with delays: $p_{\text{delay}}=10^{-4}$ , d=20 ). (a) Average proportion of heterozygotes in each patch. (b) Average number of individuals with the original cycle length of a given patch

These results are in line with the observations made from the coemergence trials, that is, rare but long delays favor prime‐numbered cycles. To gain further insight on the mechanism by which delays and hybridization favor prime‐numbered cycles, we used the case with no delays and random starting ages as a benchmark to calculate the average proportion of heterozygotes in each patch (starting from the time point when migration is allowed to occur up until either the original resident trait was invaded or the simulation ended). Then, we used the case with delays ( $p_{\text{delay}}=10^{-4}$ , d=20 ) to calculate for each patch the average proportion of heterozygotes between the first occurrence of a delay and invasion of a new cycle length (or until the end of the simulation, if no invasion occurred). We find that in patches where the resident cycle length was prime‐numbered, the average proportion of heterozygotes was slightly reduced after a delay occurred. In contrast, the average proportion of heterozygotes increased, sometimes many fold, in patches with nonprime‐numbered resident cycles (Figure 6a).

From the simulation data, we calculated mean emergence size and mean number of deaths due to predation for homo‐ and heterozygotes, respectively. In the benchmark case with no delays, average mortality due to predation (the ratio of mean deaths to mean emergence size) for homozygotes and heterozygotes was 5.7% and 55.2% of the emerging adult population, respectively. Similarly, in the case with delays, average mortality due to predation (between the first delay and invasion of a new trait) was 6.4% and 49.5% of the emerging adult population for homozygotes and heterozygotes, respectively. This indicates a considerable selective advantage for homozogytes. Indeed, we find that average population density decreased after the first delay occurred in patches occupied by nonprime‐numbered cycles whereas it was almost unchanged in patches occupied by prime‐numbered cycles (Figure 6b).

## 4 DISCUSSION

Our results show that if all the life cycles in the periodical cicada population share a common initial starting age, that is, all subpopulations consist of newborns at the beginning of a simulation, then prime‐numbered cycles are favored. This is because nonprime‐numbered cycles coemerge more often than prime‐numbered cycles and are therefore more negatively affected by hybridization. However, if we assume random starting ages for each subpopulation, then prime numbers have no particular advantage in terms of reduced number of coemergences and we do not observe that prime‐numbered cycles would be favored over nonprime‐numbered cycles. Rather, the population as a whole appears fairly stable such that invasions occur relatively rarely. Typically, simulations end with almost all patches retaining their original cycle length.

The scenario where the entire population shares a common starting age is not general, but rather a special case. However, since with random starting ages the local populations are relatively stable, any mechanism that would occasionally act so as to (partially) synchronize emergence within the whole population could potentially tip the balance towards prime‐numbered cycles. One such potential mechanism is environmentally induced dormancy: Many insect species are known to become dormant during periods of harsh environmental conditions (Danks, 1987) followed by a synchronized emergence after the environmental conditions became suitable, for example, even up to 30‐year delays under artificial conditions have been reported for the moth *Prodoxus y‐inversus* (Powell, 2001). This appears particularly promising, as it has been previously postulated that the evolution of the long life cycles in periodical cicadas is linked to the harsh environmental conditions encountered during the Pleistocene ice age (Toivonen & Fromhage, 2019).

Our results show that prime‐numbered cycle lengths are favored when we assume random initial ages for each subpopulation and occasionally occurring population‐wide delays in emergence, that is, environmentally induced dormancy. The delays (partially) reset the population to a similar state as in the case where all subpopulations share a common starting age. Then, nonprime‐numbered cycles tend to begin coemerging more frequently and as a result they also produce more hybrid offspring who do not share the same life‐cycle length as either parent population. We show that these hybrids have a high mortality rate due to predation as they tend to emerge in low numbers due to being asynchronous with both parent populations. Thus, hybridization has a detrimental effect on nonprime‐numbered cycles and we show that indeed their population densities tend to reduce after a delay in emergence has occurred. In contrast, we do not observe that the delays would have much of an effect on prime‐numbered cycles.

We find that short delays do not have a significant impact, but if the delays are ten or more years, then prime‐numbered cycles become more common in the population. It is possible that short delays might allow short prime‐numbered cycles to gain an advantage, but here our starting point is competition between long (10–20 year) cycle lengths. We note that, for example, a delay of ten years does not mean that the cicadas wait for an extra ten years to emerge, but rather, if their natural emergence would occur within that ten‐year interval, then the emergence is delayed until the end of the delay interval.

Natural populations of *Magicicada* are encountered in geographically contiguous broods, which emerge in different years (Lloyd & Dybas, 1966b; Williams & Simon, 1995). The differences among brood emergence years may have developed simply through random events (e.g., delays in emergence) in the past that have carried over to the present as spatial and temporal isolation of the broods has cut off their interactions. There is, however, evidence suggesting that *Magicicada* are capable of four‐year accelerations so that 17‐year cicadas may occasionally emerge already after 13 years (Lloyd & Dybas, 1966b; Lloyd & White, 1976). There is debate whether this occurs through hybridization (Cox & Carlton, 1988, 1991, 2003) or phenotypic plasticity (Marshall & Cooley, 2000; Martin & Simon, 1988, 1990; Simon et al., 2000). In any case, it appears that the split to 13‐ and 17‐year cycles is relatively recent (Sota et al., 2013). In this paper, our goal has been to investigate the causes of the initial selection for prime‐numbered life cycles in *Magicicada* over nonprime cycles. Indeed, our results suggest the plausibility of hybridization having been an important factor in selection for prime‐numbered cycles. The potential mechanisms causing life cycle switching is not something we have considered here.

The individual‐based model (IBM) that we have used to simulate cicada life cycles is similar to previous work (Toivonen & Fromhage, 2018, 2019). However, one notable difference is our modeling of nymph competition within the ground. Previously, Toivonen and Fromhage (2018, 2019) used simple continuous density‐dependent mortality, but here we employ a so‐called safe‐site model (Geritz, 1995; Skellam, 1951). The main difference is that in the previous work all nymphs were equal competitors irrespective of age or size. The safe site model is the opposite: established nymphs do not compete against each other and are superior to newborns. Equal competitive ability irrespective of age and size seems unrealistic and can be avoided by making competition more asymmetric, which is accomplished here with the safe site model. Further, we have found that the density‐dependent model favors short cycles over long ones: if all nymphs irrespective of age are equal competitors, then short cycles enjoy a higher chance of survival until emergence. Thus, for the evolution of long life cycles to be possible, competition among nymphs likely includes some form of age or size dependency. Alternatively, if competition among nymphs were equal, then long cycle lengths would need to have a higher fecundity compared with short cycles in order to be competitive.

The second main difference between our model here and previous models is that here we have embedded the IBM in a structured population framework. This choice reflects the hypothesis that multiple synchronous life cycles evolved in *Magicicada* in isolation during a glacial period. Then, our goal was to investigate whether competition between different cycles would favor prime‐numbered cycle lengths once migration between previously isolated populations became possible. Further, we find that competition of multiple traits within a single patch does not favor prime‐numbered cycle lengths. We include simulation results in the Appendix 1 showing that prime‐numbered cycles are not favored in a single‐patch model even when we assume that the initial population consists of newborns only. This is because in a single patch coemerging subpopulations are entirely mixed together so that the resulting heterozygote population is typically as big or bigger than the original homozygote populations (Hardy–Weinberg principle) and therefore they are not selected against due to low population size. On the other hand, in the spatially structured population setting, migrants enter new patches in low numbers and thus the resulting heterozygote populations are also small and selected against when they emerge alone. Then, hybridization acts as an extra source of mortality and avoiding it is beneficial. Further, we find that when a large number of different life cycles compete in a single patch, then the yearly emergence size of the population quickly becomes roughly constant (with some natural variation). Then, there is no selection for any particular cycle length, until stochastic variations in a small population lead to some emergence frequency to grow above others, and then individuals in synchrony with this cycle length become selected for.

We also find that the hybridization assumption is important for our results. As a control experiment, we ran simulations of the IBM assuming clonal reproduction. We find that generally no cycle performs better than others in this context, except that the 20‐year cycle dominates the 10‐year cycle (see Appendix 1).

As a further control experiment (results not shown), we performed simulations incorporating a higher number of patches (20 patches), where each patch had initially a randomly chosen cycle length (with a randomly chosen initial age). Here, we used $p_{\text{delay}}=10^{-3}$ and d=20 . We find that typically the cycle length that initially has the highest number of occupied patches is also the one that eventually drives all others to extinction (this occurred 25 times out of 30 simulations). The mean proportion of prime‐numbered individuals was slightly larger at the end of simulations compared with their mean proportion at the beginning of simulations (0.43 and 0.36, respectively). However, the difference was fairly small. The general success of the initially dominant cycle length indicates that once a given cycle becomes numerically superior to others, it has an ecological advantage irrespective of whether it is prime or nonprime. We suspect this is because a larger population size (across the entire structured population) increases the chances of dispersal to other patches at opportune moments, when those patches happen to have a temporarily reduced population size and thus more available space for invaders to occupy. Also, this numerical superiority increases chances of successful reinforcement (via migration) of the initially low‐density invaders. In any case, the focus of this paper was to demonstrate that hybridization is a potential mechanism responsible for the prime‐numbered life cycles of *Magicicada*, which we have demonstrated in a simplified (and “fair”) setting. For possible future work, it could be interesting to study how different combinations of life cycles and spatial structures might affect the development of prime‐numbered cycles, but that goes beyond the scope of this work.

Overall, we have performed a number of checks to investigate the sensitivity or our results to different modeling assumptions. However, in a complex model such as ours here, it is always possible to quibble about the details. There are many aspects of the *Magicicada* life cycle that are not fully understood and thus we have made a number of modeling assumptions. Further empirical study may in the future either validate or reject those assumptions. But ultimately, all models are abstractions, and good models are those that provide useful insights. In this paper, we highlight the potential roles of external abiotic disturbances and hybridization as key ingredients of the evolution of prime‐numbered life cycles in *Magicicada*. Indeed, our results act as a demonstration of the plausibility of this argument.

## CONFLICT OF INTERESTS

The authors declare no competing interests.

## AUTHOR CONTRIBUTION

Jaakko Toivonen: Conceptualization (equal); Formal analysis (lead); Investigation (lead); Methodology (equal); Software (lead); Writing‐original draft (lead); Writing‐review & editing (equal). Lutz Fromhage: Conceptualization (equal); Formal analysis (supporting); Funding acquisition (lead); Methodology (equal); Supervision (lead); Writing‐review & editing (equal). 

## Supporting information

Supplementary Material

## Figures and Tables

**FIGURE A1:**
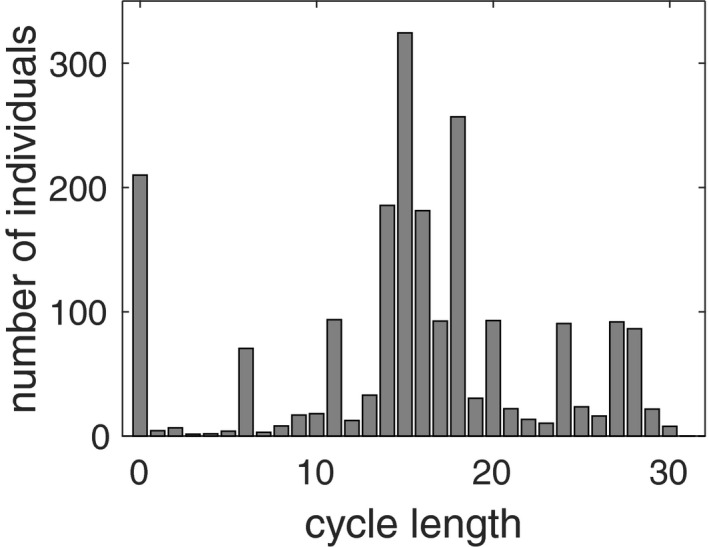
Average number of individuals for each cycle length at the end of 30 independent simulations of the IBM with only one single patch shared by all cycles. Here 0 refers to the size‐based emergence trait. The initial population consists of newborns only. At the end of the simulations, average proportion of prime‐cycle individuals is 15%. Eighteen simulations ended with one cycle length having invaded the whole population with all other cycles extinct. Parameters used were otherwise as in Table A1 except: initialPopulationsize = 2,200, patches = 1, delayProbability = 0, noMutationUntilRound = 0

## Data Availability

Codes and simulation data for the IBM and codes pertaining to the calculations in the section “Coemergence probabilities” are available at a Dryad repository: https://doi.org/10.5061/dryad.kd51c5b2r.

## Appendix 1

### IBM WITH *p*_delay_ = 10^−1^

In this case, a closer look at the population dynamics shows that all the cycles are coemerging most of the time (due to the frequent delays) and the proportion of hybrids in each patch quickly becomes very high. Then, the population in each patch becomes mixed with all cycle lengths and each cycle has roughly an equal proportion of the patch population. In this situation, all cycles have low population density and it becomes beneficial to coemerge with as many other individuals as possible, to avoid predation (alternatively, it may be easier for offspring to establish when most of the population has emerged). Then, cycle lengths less or equal to d+1 benefit from the fact that they always coemerge after a delay has occurred. On the other hand, considering cycle lengths less or equal to d+1 , the longer cycle lengths have a smaller probability of emerging between delays and are thus advantageous.

### SINGLE PATCH

Here, we model competition between age‐based emergence traits with cycle lengths from 10 to 20 in a single patch. The initial population consists of an equal number of individuals of each cycle length and each individual is a newborn established in a feeding cell. We perform 30 independent simulations assuming no delays occur. The results are summarized in Figure A1. The results show a decrease in the proportion of prime‐numbered cycles from an initial 4/11 ≈36% to 15%. Even though the initial population consists of cycle lengths between 10 and 20, we see multiple instances where cycles outside of this range evolve, with both shorter and longer cycles possible. Further, we see that size‐based emergence evolves as well, but this may be a transient feature (no simulation ends with only size‐based individuals in the population). We observe that typically the population exhibits initially relatively constant annual emergence sizes and while this remains true, size‐based emergence may evolve as the timing of emergence is not consequential. However, if some cycle length begins to dominate in the population, then size‐based emergence becomes selected against.

The appearance of the size‐based emergence trait is related to a low predator density relative to the cicada population density in the patch (predatorDensity = 5, initialPopulationSize = 2,200) and the average number of adult cicadas that emerge yearly. We performed further tests with higher predator densities (predatorDensity equal to 30 and 55) and found that size‐based emergence did not evolve. Also, prime numbered cycles did not perform significantly better than would be expected by a random process: The proportion of individuals with a prime numbered cycle was 43% and 27% of the population with predatorDensity equal to 30 and 55, respectively (with an initial proportion of 36% at the start of simulations). In the corresponding case with spatial structure (11 patches, no delays, initial population consists of newborns only), the final proportion of prime cycles was 74%.

### CLONAL REPRODUCTION

As a control experiment, we performed additional simulations where the only difference in the IBM is that reproduction is clonal (we used $p_{\text{delay}}=10^{-3}$ and d=20 ). Interestingly, we found that the cycle length 20 was dominant: 17 runs out of 30 ended with one trait having more than 95% of the population, and in all of these cases the cycle length 20 was selected. A closer look at the population dynamics revealed that the 20 year cycle was always driving the 10 year cycle to extinction and then it proceeded to eventually invade the rest of the population through numerical superiority. Another set of experiments with only cycle lengths 10 to 19 in the initial population showed that no trait was selected for over the others (almost all simulations ended with all the original cycles holding their initial patch).

If a 20‐year cycle individual gets established in a 10‐year cycle patch and is synchronized with the main population of the patch, it has a selective advantage: every time it emerges it has the same chance of establishing new offspring as the resident population, but when the 10‐year cycle individuals emerge the next time, the 20‐year cycle offspring still remain in the ground and reduce the available space for new offspring. The 20‐year cycle individuals thus temporarily reduce the effective carrying capacity of the patch for the 10‐year cycle, but it pays no penalty itself as it always emerges at the same time as the 10‐year cycle individuals. In contrast, when sexual reproduction is assumed, both traits suffer equally from hybridization, when they coemerge.

**TABLE A1:** Essential simulation parameters, their typical values (or range of values) and short description

Variable	Value	Description
initialPopulationSize	200	Carrying capacity of each patch ( N ).
patches	(1,2,…,10,11)	Vector that designates a unique integer for each patch.
targetSize	5	Body size required before emergence is possible.
mutationProbability	0.001	Probability that a given allele mutates in reproduction.
migrationProbability	0.2	Probability that a given adult migrates from its nymphal patch.
noMutationUntilRound	2,000	Parameter defining the length of the initialization time period.
delayProbability	0–0.1	Probability that emergence is not possible in a given year.
delayEnd	0–20	Number of years of a delay (years).
timeMax	52,000	Number of years simulated in each run.
growthMean	0	Log‐normal mean growth rate (*µ*).
growthStandardDeviation	0.3	Log‐normal standard deviation of growth rate ( σ ).
predatorAttackRate	1	Predator attack rate to catch emerging adult cicadas.
predatorDensity	5	Population density of predators (identical for each patch).
predatorHandlingTime	1	Average time predators spend processing caught cicadas before resuming predation.
